# Potentiality of *Bacillus subtilis* as biocontrol agent for management of anthracnose disease of chilli caused by *Colletotrichum gloeosporioides* OGC1

**DOI:** 10.1007/s13205-013-0134-4

**Published:** 2013-04-17

**Authors:** N. Ashwini, S. Srividya

**Affiliations:** Department of Microbiology, Centre for PG Studies, Jain University, 18/3, 9th Main, Jayanagar 3rd Block, Bangalore, 560011 India

**Keywords:** *B. subtilis*, Mycolytic enzymes, Antagonism, *C. gloeosporioides*, UV mutagenesis

## Abstract

A soil bacterium, *Bacillus subtilis*, isolated from the rhizosphere of Chilli, showed high antagonistic activity against *Colletotrichum gloeosporioides* OGC1. A clear inhibition zone of 0.5–1 cm was observed in dual plate assay. Microscopic observations showed a clear hyphal lysis and degradation of fungal cell wall. In dual liquid cultures, the *B. subtilis* strain inhibited the *C. gloeosporioides* up to 100 % in terms of dry weight. This strain also produced a clear halo region on chitin agar medium plates containing 0.5 % colloidal chitin, indicating that it excretes chitinase. The strain also produced other mycolytic enzymes—glucanase and cellulase, demonstrated by a clear zone of hydrolysis of yeast cell wall glucan (YCW 0.1 % v/v) and carboxymethylcellulose (CMC 0.1 % v/v). In liquid cultures, the strain showed appreciable levels of chitinase, glucanase and cellulase activities and hydrolytic activity with *C. gloeosporioides* OGC1 mycelia as the substrate. The role of the *B. subtilis* strain in suppressing the fungal growth in vitro was studied in comparison with a UV mutant of that strain, which lacked both antagonistic and hydrolytic activity. The mycolytic enzyme mediated antagonism of *B. subtilis* was further demonstrated by heat inactivation (70–100 °C), treatment with trypsin and TCA of the crude enzyme extract which lacked antifungal property also. Treatment of the chilli seeds with *Bacillus* sp. culture showed 100 % germination index similar to the untreated seeds. The treatment of the seed with co-inoculation of the pathogen with *Bacillus* sp. culture showed 65 % reduction in disease incidence by the treatment as compared to the seed treated with pathogen alone (77.5 %).

## Introduction

Rhizosphere bacteria are excellent agents to control soil-borne plant pathogens. Bacterial species such as *Bacillus*, *Pseudomonas*, *Serratia* and *Arthrobacter* have been proved in controlling the fungal diseases (Joseph et al. 2007) Earlier reports showed that microorganisms capable of lysing chitin, which is a major constituent of the fungal cell wall, play an important role in biological control of fungal pathogens (Yu et al. 2002; Zhang and Fernando 2004; Abdullah et al. 2008). Fungi such as *Trichoderma* and bacteria such as *Bacillus*, *Serratia* and *Alteromonas* were reported to have chitinolytic activity (Mabuchi et al. 2000; Someya et al. 2001; Wen et al. 2002; Huang et al. 2005; Viterbo et al. 2001). Non-pathogenic soil *Bacillus* species offer several advantages over other organisms as they form endospores and hence can tolerate extreme pH, temperature and osmotic conditions. *Bacillus* species were found to colonize the root surface, increase the plant growth and cause the lysis of fungal mycelia (Turner and Backman 1991; Podile and Prakash 1996; Takayanagi et al. 1991).

Chilli, *Capsicum annum* L. cultivation has existed for several 100 years as a sustainable form of agriculture in India and in many other countries. It is an annual herbaceous vegetable and spice grown in both tropical and subtropical regions. The crop is grown in almost all states of India, such as Andhra Pradesh, Maharashtra, Karnataka, Gujarat, Tamil Nadu and Orissa. India accounts for 25 % of the world’s total production of chilli (http://agropedia.iitk.ac.in/content/area-and-production-chilli-world-2008-09).

The crop is a significant source of income making India the world’s single largest producer and exporter to the USA, Canada, UK, Saudi Arabia, Singapore, Malaysia, Germany and many more countries across the world. The sustainability of chilli-based agriculture is threatened by a number of factors. Main biotic stresses such as bacterial wilt, anthracnose, viruses and several insect pests have been reported to impair the crop productivity (Isaac 1992).

The genus *Colletotrichum* and its teleomorph *Glomerella* contain an extremely diverse number of fungi including both plant pathogens and saprophytes. Plant pathogenic species are important worldwide, causing pre- and post-harvest losses of crops (Bosland and Votava 2003). These fungi cause diseases commonly known as anthracnose of grasses, legumes, vegetables, fruits and ornamentals. The disease can occur on leaves, stems and fruit of host plants (Sutton 1992). Anthracnose disease is a major problem in India and one of the more significant economic constraints to chilli production worldwide, especially in tropical and subtropical regions (Than et al. 2008).

Economic losses caused by the disease are mainly attributed to lower fruit quality and marketability. Although infected fruits are not toxic to humans or animals, severely affected fruits showing blemishes are generally considered unfit for human consumption. This is because the anthracnose causes an unpleasant colour and taste in chilli products. Management of the disease under the prevailing farming systems in India has become a recurrent problem to chilli growers (Thind and Jhooty 1985).

Effective control of anthracnose disease involves the use of one of, or a combination of, the following: resistant cultivars, cultural control and chemical control. The intensive use of fungicides has resulted in the accumulation of toxic compounds potentially hazardous to humans and the environment, and also in the build-up of resistance of the pathogens. In view of this, investigation and the application of biological control agents (BCAs) seems to be one of the promising approaches (Cook 1985). Biocontrol involves the use of naturally occurring non-pathogenic microorganisms that are able to reduce the activity of plant pathogens and thereby suppress diseases. Hence, controlling this pathogen using biocontrol agents will help in enhancing the yield of the crop.

*B. subtilis* JN032305, isolated from chilli rhizosphere produced appreciable levels of three mycolytic enzymes—chitinase, glucanase and cellulase and showed broad spectrum antagonism against potent bacterial and fungal phytopathogens (Srividya et al. 2012). The production of all three enzymes of this strain have been optimised using Plackett-Burman approach (Ashwini and Srividya 2012a, b). The objective of the present study was to ascertain the concerted role of these three mycolytic enzymes (chitinase, β1,3-glucanase and cellulase) mediated antagonism of *B. subtilis* against *C. gloeosporioides.*

## Materials and methods

### Isolation of rhizospheric *Bacillus* sp.

Chilli rhizosphere soils were collected from in and around Bangalore and *Bacillus* sp. were isolated by soil dilution method. The dilutions were heat treated at 80 °C for 20 min to ensure that only heat resistant strains remained. The different isolates obtained were screened for chitinase production based on the halo produced on plates with minimal salts medium amended with chitin (1 % chitin) (Cook 1985). The *Bacillus* sp., thus, obtained were maintained on Nutrient agar (NA) amended with chitin (1 %).

### Morphological and phenotypic characterization of *Bacillus* sp.

This strain was characterised morphologically and biochemically by following Bergey’s Manual of Systematic Bacteriology (Sneath 1986) and was found to be a *Bacillus* sp. It was grown and maintained on NA at 30 °C. Polymerase chain reaction (PCR) was performed to amplify a partial 16S rRNA gene of the bacteria, and partial 16S rDNA sequencing was used to assist in the identification of the isolate. Isolation of genomic DNA, PCR amplification and sequencing of PCR product for analysis of 16S rRNA were conducted according to Marchesi et al. (1998). A similarity search for the nucleotide sequence of 16S rRNA of the test isolate was carried out using a blast search at NCBI (Altschul et al. 1990).

### Preparation of colloidal chitin

Colloidal Chitin was prepared from crab shell chitin powder (Roberts and Selitrennikoff 1988). A few modifications were made as described: 10 g of chitin powder was added slowly into 100 mL of concentrated HCl under vigorous stirring for 2 h. The mixture was added to 1,000 mL of ice-cold 95 % ethanol with rapid stirring and kept overnight at 25 °C and then stored at −20 °C until use. When needed, the filtrate was collected and washed with 0.1 M phosphate buffer (pH 7) until the colloidal chitin became neutral (pH 7) and used for further applications.

### Phytopathogens and chilli seeds

*Colletotrichum gloeosporioides* (OGC1) and chilli seeds (Arka Shweta variety) were obtained as a kind gift from IIHR, Hessarghatta, Bangalore.

### Dual plate assay

The fungal growth inhibition capacity of *Bacillus* sp. strains was determined (Huang and Hoes 1976). A few modifications were made to suit the need. One 5-mm disc of a pure culture of the pathogen was placed at the centre of a Petri dish containing PDA. The *Bacillus* sp. was inoculated at two opposing corners. Plates were incubated for 72 h, at 28 °C, and growth diameter of the pathogen was measured and compared to control growth, where the bacterial suspension was replaced by sterile distilled water. Each experiment using a single pathogen isolate was run in triplicate. Results were expressed as the means of the percentage of inhibition of growth of the corresponding pathogen isolate in the presence of any of the strain of *Bacillus* sp.

Percent inhibition was calculated using the following formula:

### Detection of extracellular hydrolytic activity

Plates with minimum salts medium (MSM) containing (1 % w/v) carboxy methyl cellulose (CMC) was prepared. The *Bacillus* sp. was spot inoculated in the centre of the plate. After an appropriate incubation period at 30 °C for 48 h, the agar medium was flooded with an aqueous solution of Congo red for 15 min. The Congo red solution was then poured off and plates containing CMC were visualised for zones of hydrolysis (Shanmugaiah et al. 2008; Moataza 2006; Teather and Wood 1982) detecting β-1,4 cellulase. Yeast Glucan containing plates (Chen et al. 1995) was used to detect β-1,3 cellulase activity. MSM with (1 % v/v) yeast cell glucan was prepared and spot inoculated with the isolate. Development of a clear zone surrounding the colony indicated enzyme production.

### Assay of hydrolytic enzymes

#### Chitinase enzyme assay

Chitinase activity was measured with colloidal chitin as a substrate. The culture broth was centrifuged and enzyme solution of 1 ml was added to 1.0 ml of substrate solution, which was made by suspending 1 % of colloidal chitin in Phosphate buffer (pH 7.0). The mixture was incubated at 50 °C for 30 min. 1 ml of DNS was added and incubated at 100 °C in boiling water bath. The amount of reducing sugar produced in the supernatant was determined by dinitrosalicylic acid method (DNS) (Miller 1959). One unit of chitinase activity was defined as the amount of enzyme that produced 1 μmol of reducing sugars per min (Annamalai et al. 2008).

#### Cellulase β-1,3 assay

The specific activity of β-1,3-cellulase was determined by measuring the amount of reducing sugars liberated using dinitrosalicylic acid solution (DNS) (Annamalai et al. 2008). The culture broth was centrifuged and enzyme solution of 1 ml was added to 1.0 ml of substrate solution which contained 1 ml of yeast cell wall extract (YCW 1 % v/v). The mixture was incubated in a water bath at 50 °C for 30 min and the reaction was terminated by adding 1 ml of DNS solution and incubated in boiling water bath for 10–15 min till the development of the colour of the end product. Reducing sugar concentration was determined by optical density at 540 nm (Gadelhak et al. 2005).

#### Cellulase β-1,4 assay

The specific activity of β-1,4-glucanase was determined by measuring the amount of reducing sugars liberated using dinitrosalicylic acid solution (DNS) (Annamalai et al. 2008). The culture broth was centrifuged and enzyme solution of 1 ml was added to 1.0 ml of substrate solution which contained 1 ml of carboxy methyl cellulose solution (CMC 1 % v/v). The mixture was incubated in a water bath at 50 °C for 30 min and the reaction was terminated by adding 1 ml of DNS solution and incubated in boiling water bath for 10–15 min till the development of the colour of the end product. Reducing sugar concentration was determined by optical density at 540 nm (Moataza 2006).

### Induction with fungal mycelium

The *Bacillus* sp. was grown on Nutrient broth supplemented with dead fungal mycelium (*Colletotrichum gloeosporioides* OGC1) as inducer for enzymes production at a concentration of 1.0 % and dispensed in Erlenmeyer flasks (250 ml), each flask containing 50 ml of medium. The flasks were autoclaved and inoculated with 1.0 ml of a pre-cultured *Bacillus* sp. culture. The culture was incubated in a shaker (120 rpm.), at 28 ± 2 °C. Aliquots from the flask were analyzed daily for chitinase and cellulases (β-1,3 and β-1,4) for a period of 5 days (Moataza 2006).

### Hydrolytic assay

#### Preparation of hyphal wall

The pathogenic fungal culture (*C. gloeosporioides*) was inoculated into 50 ml of PDB broth and incubated at 30 °C for 5 days under shaking conditions. After incubation, the mycelia were collected by filtration. The mycelia were thoroughly washed with autoclaved distilled water and homogenised on ice, with a homogenizer for 5 min. The mycelia suspension was centrifuged at 10,000 rpm for 20 min at 4 °C (Remi: C 24). The pellet was resuspended in sodium phosphate buffer (0.1 M, pH 7.0). This preparation was used as substrate for the hydrolytic assay (Moataza 2006).

#### Hydrolytic activity of *B. subtilis* (wild type and mutants) culture filtrate

For assessing the hydrolytic activity, the reaction mixture (1 ml) containing 1 mg/ml of fungal mycelia with 1.0 ml of crude enzyme (from *B. subtilis* grown on NB + CMC) was incubated at 30 °C for 24 h. The released total reducing sugars (Miller 1959) in control and treated fungal cell wall was estimated using the DNS method.

### Dual culture method

The differences in dry weights between the fungal cultures grown with *B. subtilis* strain or the control culture grown without any bacterium were recorded according to Broekaert et al. 1990. For this, 48 h grown dual cultures were passed through the pre-weighed Whatman No 1 filter paper. It was dried for 24 h at 70 °C and weights were measured (Saleem and Kandasamy 2002).

### Microscopy

The fungal culture grown on NA agar plate without any bacterial culture served as control. The damage caused by the bacterium to the fungal mycelium by dual plate assay was studied microscopically. The mycelium along with the agar disc present in the inhibition zone and control mycelium was taken, stained with lactophenol cotton blue and observed under a Nikon Trinocular microscope (Saleem and Kandasamy 2002).

### UV mutagenesis

To characterise the antagonistic mechanism by this bacterium, a mutant of this bacterium was developed, which lost its antagonistic activity. UV mutagenesis of *B. subtilis* was carried out following the procedure of Miller (1992). During UV mutagenesis, the log phase culture was exposed to short wavelength UV light (280 nm, Philips TUV 30 W, G3018, Holland) from a distance of 30 cm for various time intervals (for 0.1 % UV survivors). From the serial dilutions of the mutagenized culture, 0.1 ml was plated on NA plates for isolated mutant colonies. The isolated colonies were further screened for the loss of antifungal activity using dual plate assay. The mutants were also screened for mycolytic enzyme activity and hydrolytic activity with the pathogen mycelium.

### Sensitivity of the culture supernatant of wild type *B. subtilis* to proteolytic enzymes, TCA and heat

The crude supernatant (1 mL) was subjected to treatments for 1 h at 37 °C (for enzymes) or room temperature (for TCA). The proteolytic enzymes (Sigma) were used at a final concentration of 1 mg ml^−1^ in 10 mmol^−1^ potassium phosphate buffer, pH 7.0. The crude supernatant in buffer without enzymes as well as the enzyme solutions was exposed to the same conditions. For the heat treatment, the preparations were incubated at 70, 80 and 90 °C and autoclaving for 20 min. Antifungal activities were checked before and after all treatments on a test plate made with *C. gloeosporioides* as mentioned earlier (Tendulkar et al. 2007).

### Seed testing

Germination efficiency and antagonism of the *Bacillus* sp. against *C. gloeosporioides* was checked on chilli seeds in vitro. The water agar plates were seeded with the following: Set 1-Seeds control-plain seeds coated with carboxy methyl cellulose (CMC); Set 2- Seeds coated with CMC and *C. gloeosporioides* spores; Set 3- Seeds coated with CMC and *Bacillus* sp. culture and Set 4- Seeds coated with CMC and both *C. gloeosporioides* spores and *Bacillus* sp. culture. Chilli seeds were surface sterilised successively with sterile distilled water and 0.1 % HgCl_2_. To remove the residual HgCl_2_, the seeds were washed with sterile distilled water. The isolate was inoculated into NB medium and incubated for 24 h at 30 °C (Moataza 2006). *C. gloeosporioides* was inoculated onto PDA plates and incubated at 28 °C for 3–4 days. The above three sets of treated seeds were seeded onto 1 % water agar plates. Plain CMC coated seeds on water agar were used as control. The four sets were monitored regularly for germination and growth. After 1 week, the sets were observed for germination and biocontrol against *C. gloeosporioides* coated seeds by the isolate.

### Reproducibility

All the experiments were done in triplicates and the values represented statistically are in ANOVA form.

## Results and discussion

15 chilli rhizosphere soils samples were collected from in and around Bangalore, heat treated and plated on chitin amended plates. Out of 15 samples plated, 9 chitinolytic colonies were isolated based on the clearance zones that they formed.

### Dual plate assay

**Fig. 1:**
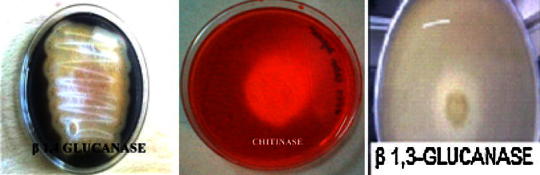
Plates showing zone of hydrolysis for β1,4-glucanase; chitinase and β 1,3 glucanase by WT *B. subtilis*

These chitinolytic bacteria were subjected to dual plate assay with nine different chilli fungal pathogens, one particular isolate designated as isolate two showed inhibition of all the nine pathogens and this was chosen for further work. The selected isolate showed broad spectrum antagonism against *Alternaria* (3) spp. (55 %), *C. gloeosporioides* (57 %), *Phytophthora capsici* (55 %), *Rhizoctonia solani* (42 %*), Fusarium solani* (42 %), *Fusarium oxysporum* (40 %) and *Verticillium* sp. (36 %), the range of percentage inhibition varied from 40 to 62 (Table 1). The isolate was checked for other hydrolytic enzyme production and was found to produce both Cellulase β-1,3 and β-1,4 on the basis of clearance zones produced on yeast glucan plates as well as CMC plates, respectively (Fig. 1).

**Table 1:** Results of the dual plate assay

Pathogens	Inhibition (%)
*C. gloeosporioides* (OGC1)	57 ± 0.901^d^
*A. brassicae* (OCA1)	57 ± 2.685^a^
*A. brassicicola* (OCA3)	53 ± 1.443^a^
*A. alternata* (OTA36)	45 ± 1.01^c^
*P. capsici* (98-01)	62 ± 0.935^c^
*Verticillium* sp.	52 ± 2.049^c^
*F. solani*	41 ± 1.527^b^
*F. oxysporum*	40 ± 1.607^e^
*R. solani*	56 ± 5.204^f^

Results are the mean of three replicates ± SD. In columns, values with the same letters are not significantly different (*P* < 0.05 Duncan test)

### Strain identification

**Fig. 2:**
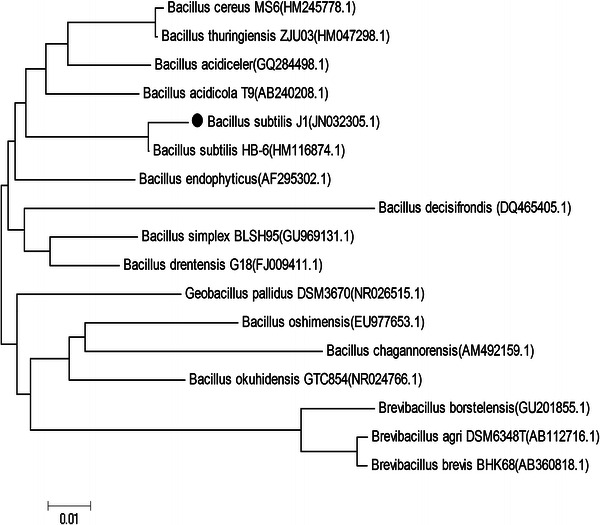
Phylogenetic tree constructed with the 16S rDNA sequences

This strain was characterised morphologically and biochemically by following Bergey’s Manual of Systematic Bacteriology and was found to be a *Bacillus* sp. It was grown and maintained on NA at 30 °C. The isolate upon Gram’s staining was identified as a gram positive, spore forming rod. The isolate was motile and answered positive for catalase, nitrate reduction, Voges Proskauer, starch hydrolysis and growth on 6.5 % NaCl medium and negative for parasporal crystal formation and citrate utilisation, hence, was tentatively identified as *Bacillus* sp. Further, 16SrDNA sequencing identified the isolate to be *B. subtilis* and the Gene-Bank accession no. for the nucleotide sequence is JN032305 (Fig. 2) (Ashwini et al. 2012).

### UV mutagenesis

**Fig. 3:**
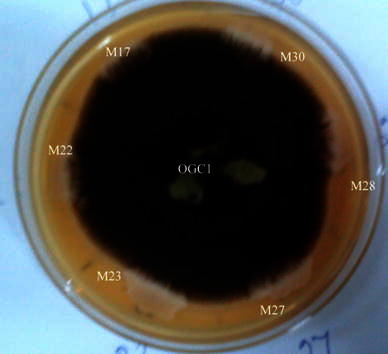
*Bacillus* strain mutants M17, M22, M23, M27, M28 and M30 not showing inhibition to *Colletotrichum* on PDA

To characterise the antagonistic mechanism by this bacterium, a mutant of this bacterium was developed, which lost its antagonistic activity. Out of 61 putative mutant colonies tested, six showed no antagonistic property against *C. gloeosporioides* (Fig. 3). These mutant isolates also did not grow over the mycelial mat and were named as M17, M22, M23, M27, M28 and M30.

### Microscopy

**Fig. 4:**
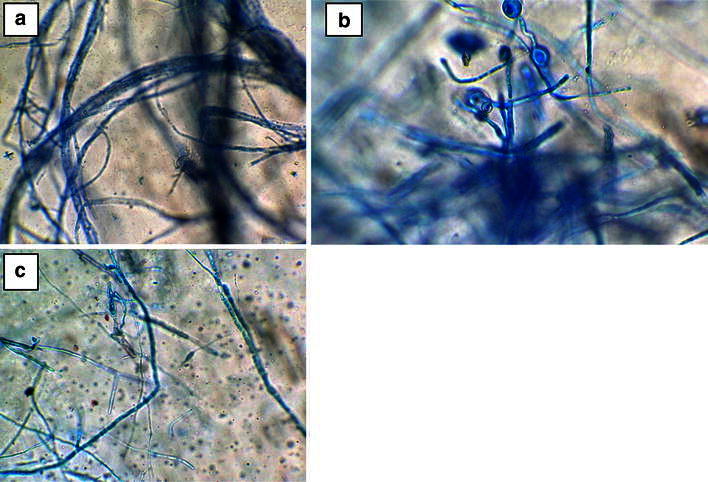
Light microscopic observations of mycelium inhibited by BC2 strain (1,000×). Mycelium of *Colletotrichum***a** grown on PDA (control), **b** present in the inhibition zone, when grown with *Bacillus* strain BC2 on PDA, **c** grown with mutant strains on PDA medium

The fungal mycelium grown with *Bacillus* BC2 culture showed damage, swelling and distortions as compared to the control (Fig. 4a, b). The mycelium which grew with *Bacillus* BCUVM cultures and the control mycelium which was not grown with any bacterial culture did not show these abnormal features (Fig. 4c). This clearly indicates the mycolytic activity of the *Bacillus* BC2 culture. A similar observation has been made in the antagonism of *Arthrobacter* sp. to *Fusarium* sp. (Barrows-Broaddus and Kerr 1981). Similarly, Podile and Prakash (1996) reported the lysis and dissolution of fungal mycelium of *Aspergillus niger* by *B. subtilis* AF1 strain.

### Mycolytic enzyme activities

**Fig. 5:**
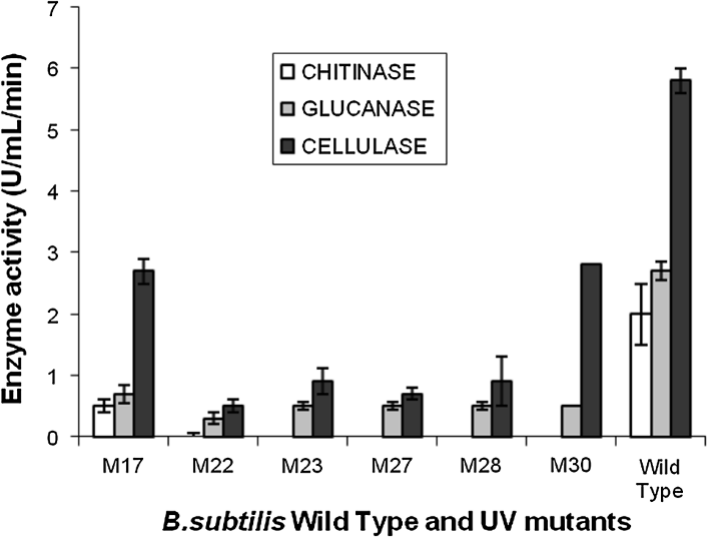
Comparison of mycolytic enzyme activities of the mutants and the wild strain BC2

**Fig. 6:**
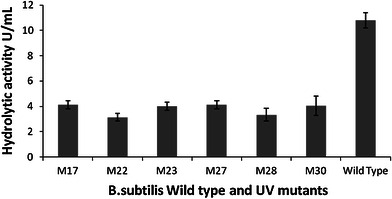
Comparison of hydrolytic activities of the mutants and the wild strain BC2. Activities of all three enzymes on day 1 by WT *B. subtilis* is represented

The induction profile of the *Bacillus* sp. was checked with autoclaved *C. gloeosporioides* mycelium used as the carbon source in the medium. Data represented in (Fig. 5) showed that the lysis of dead mycelia of *C. gloeosporioides* was very efficient by the *Bacillus* sp. Appreciable levels of all the three enzymes were observed in the presence of the autoclaved mycelia—chitinase peaked on day 1 (2.84 U/mL) and gradually decreased (2 U/mL by day 2 and 1.4 U/mL by day 3); β-1,3 cellulase was detected from day 1 (2.8 U/mL) which peaked on day 2 (3.2 U/mL) followed by a gradual decrease (2.7 U/mL by day 3 and 1 U/mL by day 4) and β-1,4 cellulase was also detected from day 1 (6 U/mL) and increased to a maximum of 13.21 U/mL by day 3 followed by a gradual decrease on day 4 (8 U/mL) suggesting the possible role of these enzymes in antibiosis of the mycelia. Moataza 2006 also reported varied levels and types of mycolytic enzymes by different *Pseudomonas* strains with different pathogens such as *P. capsici* and *R. solani*. Further, the mutants were studied for their mycolytic enzyme activities under shake flask conditions. All the mutants showed significant loss of all three mycolytic enzyme activities (Fig. 5). The mutants also exhibited low levels of hydrolytic activity with *C. gloeosporioides* mycelia as compared to the wild type strain indicating clearly the mycolytic enzyme mediated antagonism of this strain (Fig. 6).

### Dual culture method

To test the antifungal activity of the *Bacillus* strain BC2, dual liquid culture method was employed. The differences in dry weights between the fungal cultures grown with BC2 strain or the mutant strains or the control culture grown without any bacterium were recorded according to Broekaert et al. (1990). There was almost 100 % reduction in dry weight of the culture grown with BC2 strain when compared to the control. There was very little reduction in dry weight of the culture when grown with the mutant strains. This clearly shows that the reduction in dry weight of the fungus when grown with the BC2 strain is due to the antifungal activity of this bacterium. The mutant strains which had lost the antifungal activity could not reduce the dry weight of the fungus.

### Sensitivity of the culture supernatant of *B. subtilis* to proteolytic enzymes, TCA and heat

The sensitivity of the WT crude culture filtrate of *B. subtilis* BC2 was tested with TCA, heat and proteolytic enzymes. The results revealed that the activity was not preserved. Further, when such treated extracts were subjected to antagonism assay, the extract had lost its antagonistic property which supported the mycolytic enzyme mediated antagonism of the fungal pathogen.

### Seed bacterization

Treatment of the chilli seeds (Arka variety, obtained as a kind gift from IIHR, Bangalore) with *Bacillus* sp. culture showed 100 % germination index similar to the untreated seeds (Table 2). The treatment of the seed with co-inoculation of the pathogen with *Bacillus* sp. culture showed 65 % reduction in disease incidence by the treatment as compared to the seed treated with pathogen alone (77.5 %). Kamil et al. (2007) reported that the seed coat treatment of sunflower seeds with *B. licheniformis* induced high reduction in percentage of infection of *R. solani* damping off (from 60 to 25 %) as compared with the pathogen alone. Our observations also comply with these reports.

**Table 2:** Effect of seed treatment with effective *B. subtilis* on *C. gloeosporioides*

Treatments	Infection %	Germination %
Control	0	90
Seeds with pathogen	77.50	1
Seeds with *B. subtilis*	0	100
Seeds with Pathogen + *B. subtilis*	12.50	85

### Statistical analysis

The analysis of variance (ANOVA) has been performed for all three mycolytic enzymes and hydrolytic activity by the wild type and mutants of *B. subtilis. P* value was found to be very low at both *P* = 0.05, which indicated that there is significant difference in mycolytic enzyme and hydrolytic activity between the strains (Table 3).

**Table 3:** ANOVA for mycolytic enzyme production by wildtype and mutants of *B. subtilis*

Sources of variation	Chitinase		Glucanase		Glucanase		Hydrolytic assay	
	Between	Within	Between	Within	Between	Within	Between	Within
Degrees of freedom	5	3	5	3	5	3	5	3
Sample square	13.98	0.52	11.72	0.13	44.99	0.61	31.76	2.59
Mean square	2.79	0.17	2.34	0.04	8.99	0.20	6.352	0.86
*F* value	15.92		52.75		44.01		7.34	
*P* (5 %)	**2.9**		**2.37**		**2.45**		**3.97**	

Bold values indicate significant *P* values (5 %)

In a similar study, Saleem and Kandasamy (2002) showed the role of the *Bacillus* strain BC121 in suppressing the fungal growth in vitro when studied in comparison with a mutant of that strain, which lacks both antagonistic activity and chitinolytic activity. Another study by Balasubramanian et al. (2010) reported that on testing the biocontrol efficacy of the mutants and wild strain against phytopathogens such as *Fusarium oxysporum*, *Bipolaris oryzae, Rhizoctonia solani* and *Alternaria* sp. by dual culture assay on PDA medium, the UV H11 mutant and adapted mutant showed increased biocontrol activity when compared to wild strain. Balasubramanian et al. (2010) further reported that the antagonism of these two mutants with *F. oxysporum, R. solani, B. oryzae* and *Alternaria* sp. were varied and could be related with lytic enzyme production with fast growing ability. However, Lorito et al. (1993) reported chitinolytic enzymes contributing to the ability of *Trichoderma sp* to act as biocontrol agents. Graeme Cook and Faull (1991) reported that high antibiotic production by two *T. harzianum* mutant strains, BC10 and BC63, increased inhibition of hyphal growth of *R. solani* and *P. ultimum*; while Papavizas et al. (1982) have shown UV-induced benomyl resistant mutant to suppress the saprophytic activity of *R. solani* more effectively than the wild strain (Papavizas et al. 1982).

## Conclusion

The selection of effective antagonistic organisms is the first and foremost step in biological control. On the basis of these studies, it is concluded that the *Bacillus* BC2 isolate is showing antagonistic property probably through the enzyme mediated lytic mechanism, which has been proved to be an effective mechanism in controlling the fungal pathogens (Chet et al. 1990). The in vitro seed bacterization studies also have revealed the success of biocontrol of the pathogen. These observations and further studies will help in developing the *Bacillus* BC2 isolates as a potential biological control agent against *C. gloeosporioides.*
